# Frequency of Preoperative Localization Techniques of Parathyroid Adenoma at King Abdulaziz University Hospital, Saudi Arabia

**DOI:** 10.7759/cureus.13550

**Published:** 2021-02-25

**Authors:** Hani Z Marzouki, Mawaddah Abdulhaleem, Linah Qasim, Ahmad Aldajani, Shaza Samargandy, Amani Alhozali, Faisal Zawawi, Mazin Merdad

**Affiliations:** 1 Department of Otolaryngology Head and Neck Surgery, Faculty of Medicine, King Abdulaziz University, Jeddah, SAU; 2 Department of Otolaryngology Head and Neck Surgery, King Fahad General Hospital, Jeddah, SAU; 3 Department of Otolaryngology Head and Neck Surgery, King Fahad Armed Forces Hospital, Jeddah, SAU; 4 Department of Otolaryngology Head and Neck Surgery, Faculty of Medicine, University of Jeddah, Jeddah, SAU; 5 Department of Internal Medicine, Faculty of Medicine, King Abdulaziz University, Jeddah, SAU

**Keywords:** parathyroid pathology, parathyroid, accuracy, radiological modalities, preoperatively, imaging

## Abstract

Background

Accurate preoperative radiological localization of parathyroid pathologies paves the way to enable less invasive surgical procedures. Results on the accuracy of the different diagnostic measures are conflicting. Also, little is known about the most common location of parathyroid lesions. This paper aims to determine the most common location of parathyroid adenoma and evaluate the diagnostic performance of radiological modalities such as ultrasonography, sestamibi scintigraphy/single-photon emission computerized tomography (SPECT), magnetic resonance imaging (MRI), and computed tomography (CT) scan for the preoperative localization of parathyroid pathologies.

Methods

This is a retrospective study. Data were collected from patients who underwent total or partial parathyroidectomy at King Abdulaziz University Hospital between January 2000 and March 2020. The parathyroid adenoma site was detected preoperatively by a radiological method and confirmed postoperatively by the histopathology report. The performance of each preoperative localizing radiological method was evaluated based on the accuracy in localizing parathyroid pathology.

Results

A total of 73 patients were included in the analysis, with females being the most common gender in the study at 64%. Only complete data files were included and incomplete data files were excluded. The most frequent mode of detecting parathyroid adenoma was a sestamibi/SPECT scan (62.5%) followed by a CT scan (50%), ultrasound (34.6%), and MRI (25%). The most common location of a parathyroid adenoma was the left side.

Conclusion

Sestamibi/SPECT is a frequent radiological method for detecting the parathyroid lesion site as compared with CT, MRI, and ultrasonography.

## Introduction

Parathyroid adenoma is one of the most common causes of primary hyperparathyroidism causing hypercalcemia. Surgical removal of the gland is the definitive treatment for parathyroid adenomas [1]. Preoperative localization of parathyroid adenoma and minimally invasive parathyroidectomy is becoming the standard care, where its success depends on accurate preoperative localization of the adenoma [1]. Moreover, accurate preoperative radiological localization of various parathyroid pathologies can change the course of operative choice, to be more localized and less invasive, as parathyroid adenoma localization using a focused approach will result in lower surgical complications such as hypocalcemia and reduced operative time [2]. A sestamibi scan is often considered the modality of choice in the efficient localization of parathyroid adenoma preoperatively while some other studies suggest that using a combined radiological approach will provide more accuracy in localization [3-5]. However, even when sestamibi was used with ultrasound, an appropriate intraoperative parathyroid hormone drop was present and up to 15% of patients had additional parathyroid tissue found on bilateral exploration [6]. Generally, each institute has its own strategy in localizing parathyroid pathology depending on the most accurate available diagnostic method. Knowing the most common site of parathyroid adenoma is important, as it could help in cases of failure of localizing the adenoma preoperatively. The purpose of our study is to determine the most common location of parathyroid adenoma and to evaluate a variety of imaging techniques within the scope of preoperative parathyroid localization.

## Materials and methods

This is a retrospective review study where data from 73 patients at King Abdulaziz University Hospital between January 2000 to March 2020 was collected. The study was ethically approved by the Institutional Review Board (IRB) of King Abdulaziz University Hospital. All patients who underwent total or partial parathyroidectomy were included while incomplete files were excluded. The lesions' sites were detected preoperatively by radiology and were confirmed postoperatively by histopathology. The performance of each preoperative localizing radiological method was evaluated based on the ability of the method to localize the parathyroid pathology site by comparing it to histopathology. Different combinations of radiological modalities were used in accordance with the guideline of the hospital. Afterward, data were analyzed using the Statistical Package for the Social Sciences (SPSS) version 23 (IBM Corp., Armonk, NY). Simple descriptive statistics were used to define the characteristics of the study variables by counts and percentages for the categorical and nominal variables while continuous variables are presented by mean and standard deviations. To establish a relationship between categorical variables, we used a chi-square test. Lastly, a conventional p-value of <0.05 was the criterion to reject the null hypothesis.

## Results

A total of 73 participants were included in this study. The youngest participant was 15 years old while the oldest was 86 years old with a mean age of 45.6 ± 16.9 years. The minimum parathyroid hormone level preoperatively was 8.2 pg/mL while the highest level was 449.2 pg/mL with a mean of 109.8 ± 104.6 (Table 1). Furthermore, 64% of the participants were females, with the highest proportion of participants above the age of 40. Meanwhile, 60% of participants underwent four glands exploration, 23% had four or three, and half had parathyroidectomy while only 16% had minimal invasive exploration. The highest percentage of participants (40%) were investigated using single-photon emission computed tomography (SPECT), sestamibi, and ultrasound while a lower percentage of participants were investigated using one or other combinations of radiological examinations (Table 1).

**Table 1:** Participant characteristics CT: computed tomography, MRI: magnetic resonance image, N: numbers, SD: standard deviation, SPECT: single-photon emission computed tomography

	N	Minimum	Maximum		Mean		SD
Age	73	15	86		45.6		16.9
Parathyroid hormone (preoperatively)	72	8.2 pg/mL	449.2 pg/mL		109.8 pg/mL		104.6 pg/mL
				Frequency		Percentage	
Age group							
15-20				5		6%	
21-30				11		14%	
31-40				12		15%	
41-50				16		21%	
51-60				16		21%	
61 and more				18		23%	
Gender							
Male				26		36%	
Female				47		64%	
Type of surgery							
Minimal invasive exploration				12		16%	
4 glands exploration				44		60%	
4 or 3 and half parathyroidectomy				17		23%	
Preoperative diagnostic radiological method							
SPECT CT+ Sestamibi				11		15%	
Ultrasound				9		12%	
CT scan				4		5%	
SPECT + Sestamibi + ultrasound				29		40%	
SPECT + Sestamibi + ultrasound + MRI				2		3%	
SPECT + Sestamibi + CT scan				4		5%	
Ultrasound + MRI				1		1%	
Ultrasound + CT scan				1		1%	
Ultrasound + MRI + CT scan				1		1%	
SPECT + Sestamibi + ultrasound + CT scan				10		14%	

Out of the 73 participants, 56 underwent a SPECT scan + sestamibi, 52 had an ultrasound, 19 had a CT scan, and only four had an MRI scan. All participants had a postoperative pathological examination by the histopathology department for confirmation of the diagnosis. Regarding the site of pathology in each radiological test and its specificity, it is noted that 25% of SPECT + sestamibi cases, 25% of the MRI cases, 26% of the CT cases, and 50% of the ultrasound cases were nonspecific. On the other hand, parathyroid adenoma cases with or without hyperplasia were 44 representing 60% of the cases. The second most common pathology found was parathyroid hyperplasia representing 33% of all cases (Table 2). Regarding the site of the pathology based on the histopathological examination, 43.2% were at the right side, 45.5% at the left side, and 4.5% were ectopic lesions (Table 3).

**Table 2:** Final diagnosis of the samples

Final pathology of the frozen section	Frequency	Percentage
Parathyroid adenoma	37	50.7%
Parathyroid adenoma and parathyroid hyperplasia	6	8.2%
Parathyroid adenoma and parathyroid hyperplasia, and parathyroid cancer	1	1.4%
Parathyroid hyperplasia	24	32.9%
Parathyroid cancer	1	1.4%
Normal parathyroid tissues	1	1.4%
Not released	1	1.4%
Thyroid pathology	2	2.7%
Total	73	100.0%

**Table 3:** Distribution of parathyroid adenomas according to their site

Site of parathyroid adenoma	Frequency	Percentage
Right side	19	43.2%
Leftt side	20	45.5%
Both	3	6.8%
Ectopic	2	4.5%
Total	44	100.0%

The highest percentage of correct site identification was 62.5% in SPECT + sestamibi, followed by CT scan (50%), ultrasound (34.6%), while MRI was the lowest in correctly identifying only one of the four cases (Table 4). Furthermore, a total of 41 participants underwent both SPECT scan + sestamibi and ultrasound, out of the 10 participants (24%) had correct site identification in both exams. The probability of getting correct site identification in any of the two exams was 28/41=68%. By comparing the preoperative level of parathyroid hormone between the cases of parathyroid adenoma and which sites were correctly identified by the radiological examination and those with incorrect identification, the Mann-Whitney U test was used and showed no statistical significance between the two groups (p=0.835).

**Table 4:** Frequencies in the identification of different tests CT, computed tomography, MRI: magnetic resonance imagine, SPECT: single-photon emission computed tomography

Frequency of different tests		Correct site identification			Percentage		
SPECT scan + sestamibi		35/56			62.5%		
Ultrasound		18/52			34.6%		
MRI		1/4			25.0%		
CT		10/20			50.0%		
SPECT scan + sestamibi utilized with ultrasound							
				Correct site		Incorrect site	Total
SPECT scan + sestamibi	Correct site		Count	10		14	24
			% of Total	24.4%		34.1%	58.5%
	Incorrect site		Count	4		13	17
			% of Total	9.8%		31.7%	41.5%
Total			Count	14		27	41
			% of Total	34.1%		65.9%	100.0%

## Discussion

The present study aimed at evaluating a variety of imaging techniques within the scope of parathyroid localization to provide more insight into the best diagnostic approach, coupled with surgery. The study evaluated four different imaging modalities, namely the ultrasound, sestamibi scintigraphy/SPECT, MRI, and CT scan. Even though several past studies have demonstrated the beneficial effect of using imaging localization modalities preoperatively, our study provides more emphasis on that effect. Two frequently established imaging modalities with respect to primary hyperparathyroidism in the literature are ultrasonography and sestamibi scintigraphy/SPECT [7]. Research has taken advantage of both these modalities in terms of their accessibility and provided several insights into their effectiveness relative to the scope of this study [8].

Both the ultrasonography and sestamibi scintigraphy have a reported and overall sensitivity and a specificity level of 79% and 88%, respectively. Meanwhile, it reported a sensitivity of 74% and specificity of 68% laterality localization and a sensitivity of 72% and specificity of 50% in quadrant localization [9]. Stemming from this, the frequency for ultrasound and sestamibi scintigraphy/SPECT was at 34.6% and 62.5%%, respectively, differing greatly from the range of other reported studies of 75% and 90% [10] and 76.3% and 78.8% [4]. With regards to laterality, we reported an overall score of 43.2% on the right side and 45.5% on the left side. On the other hand, Nasiri et al. reported accuracy levels of 97% and 97.1%, respectively, for both ultrasound and sestamibi scintigraphy/SPECT [4].

However, the range of accuracies can be attributed to subjective factors, especially in the case of ultrasonography, given that the procedure is heavily dependant on the technician. Nevertheless, conclusions regarding the imaging modalities are scattered with sestamibi scintigraphy/SPECT and CT having the highest correct identification in line with recent studies reporting that 4D CT are very common in detecting parathyroid adenoma preoperatively, with detection capabilities of some occult cases as well [11-12]. Meanwhile, studies comparing ultrasonography to sestamibi scintigraphy/SPECT have found that both modalities are equal in how frequent they can predict lesions [13-14], Moreover, other studies reported that the sestamibi scintigraphy/SPECT method showed a higher accuracy rate in its prediction compared to the ultrasonography [15-16], resulting in the conflicting nature of the topic. The reported sensitivity of the standard CT has been inferior when compared to other imaging modalities in this scope, ranging between 40% and 70% [17-18] while 4D CT has been showing increasing promises perioperatively [12]. However, it should be noted that CT performs poorly if ectopic lesions are found in the lower neck [19-20]. Similarly, MRI shows a low accuracy level, measured within the range of 43%-71% [21-22], compared to a detection rate of 25% concluded from our study. Evidently, the MRI is a poor imaging modality in this area due to the small size of parathyroid lesions [23]. More studies are now exploring the accuracy levels of sestamibi/SPECT as well as 4D CT imaging. The use of these techniques are implemented when negative results are produced in other imaging methods, to which only then a CT scan/MRI showed a slight increase in localization [24]. 

The limitations of the study include relatively low sample size, not exploring all combinations of radiological combinations, and not performing accuracy, sensitivity, specificity, negative predictive value, and positive predictive value. We did not perform such testing, as we felt it will not represent the correct statistics in a low sample size.

## Conclusions

The preoperative localization of parathyroid adenoma has been seen in many gravitated studies that have and are potentially investigating the advantageous features of coupling it with surgery, which results in a more minimal and less invasive approach. Within this study, our patients benefited most from the sestamibi/SPECT modality, followed by the CT scan, ultrasound, and, finally, the MRI. More notably, sestamibi/SPECT was more frequent in detecting the lesions, which makes it an attractive procedure in order to minimize invasive surgery and lower operative time. The most common location of a parathyroid adenoma was the left side. The main limitation of the study is a small sample size, which was due to missing and incomplete data.
